# Clinical Management of Children with a Congenital Solitary Functioning Kidney: Overview and Recommendations

**DOI:** 10.1016/j.euros.2021.01.003

**Published:** 2021-02-03

**Authors:** Sander Groen in 't Woud, Rik Westland, Wout F.J. Feitz, Nel Roeleveld, Joanna A.E. van Wijk, Loes F.M. van der Zanden, Michiel F. Schreuder

**Affiliations:** a Department for Health Evidence, Radboud University Medical Center, Radboud Institute for Health Sciences, Nijmegen, The Netherlands; b Department of Pediatric Nephrology, Radboudumc Amalia Children’s Hospital, Radboud Institute for Molecular Life Sciences, Nijmegen, The Netherlands; c Department of Pediatric Nephrology, Amsterdam UMC, Amsterdam, The Netherlands; d Division of Pediatric Urology, Department of Urology, Radboudumc Amalia Children’s Hospital, Radboud Institute for Molecular Life Sciences, Nijmegen, The Netherlands

**Keywords:** Clinical management, Congenital anomalies of the kidney and urinary tract, Multicystic dysplastic kidney, Solitary functioning kidney, Unilateral renal agenesis

## Abstract

**Context:**

A congenital solitary functioning kidney (cSFK) is a common developmental defect that predisposes to hypertension and chronic kidney disease (CKD) as a consequence of hyperfiltration. Every urologist takes care of patients with a cSFK, since some will need lifelong urological care or will come with clinical problems or questions to an adult urologist later in life.

**Objective:**

We aim to provide clear recommendations for the initial clinical management and follow-up of children with a cSFK.

**Evidence acquisition:**

PubMed and EMBASE were searched to identify relevant publications, which were combined with guidelines on related topics and expert opinion.

**Evidence synthesis:**

Initially, cSFK diagnosis should be confirmed and risk factors for kidney injury should be identified using ultrasound. Although more research into early predictors of kidney injury is needed, additional congenital anomalies of the kidney or urinary tract and absence of compensatory kidney hypertrophy have repeatedly been associated with a worse prognosis. The role of voiding cystourethrography and antibiotic prophylaxis remains controversial, and is complicated by the exclusion of children with a cSFK from studies. A yearly follow-up for signs of kidney injury is recommended for children with a cSFK. As masked hypertension is prevalent, annual ambulatory blood pressure measurement should be considered. During puberty, an increasing incidence of kidney injury is seen, indicating that long-term follow-up is necessary. If signs of kidney injury are present, angiotensin converting enzyme inhibitors are the first-line drugs of choice.

**Conclusions:**

This overview points to the urological and medical clinical aspects and long-term care guidance for children with a cSFK, who are at risk of hypertension and CKD. Monitoring for signs of kidney injury is therefore recommended throughout life. Large, prospective studies with long-term follow-up of clearly defined cohorts are still needed to facilitate more risk-based and individualized clinical management.

**Patient summary:**

Many children are born with only one functioning kidney, which could lead to kidney injury later in life. Therefore, a kidney ultrasound is made soon after birth, and other investigations may be needed as well. Urologists taking care of patients with a solitary functioning kidney should realize the long-term clinical aspects, which might need medical management.

## Take Home Message

Urologists should be aware of the clinical aspects and long-term risks for their patients with a congenital solitary functioning kidney, which predisposes to kidney injury and warrants long-term follow-up. Kidney ultrasound is the preferred tool to confirm diagnosis and screen for additional urogenital anomalies. Thereafter, a yearly blood pressure and urine albumin-creatinine measurement is indicated, and additional treatments can be recommended depending on the long-term individual outcome of these patients.

## 1 Introduction

Every urologist takes care of patients with a solitary functioning kidney (SFK), and within the field of pediatric urology, for transition of care, and for adult urologists taking over the care of patients with a congenital anomaly, clear clinical management tools are needed. This overview points to the urological and medical clinical aspects and provides long-term care guidance for children with a congenital solitary functioning kidney (cSFK), which is a developmental defect with an estimated prevalence of 1 in 1500 newborns [1], [2], [3]. Annually, >5000 children are born with a cSFK in the USA and EU alone, and in most cases, a cSFK is the consequence of unilateral renal agenesis (URA) or multicystic dysplastic kidney (MCDK). Two systematic reviews estimated the prevalence of URA and MCDK to be approximately 1 in 2000 and 1 in 4300 newborns, respectively, and this appears to be stable in more recent cohorts (Supplementary Table 1) [2], [3]. More males than females are affected, and a left-sided cSFK seems slightly more prevalent (Supplementary Table 2) [2], [3].

Living with a cSFK predisposes to hypertension, proteinuria, and kidney function loss [4], [5], [6]. The magnitude of the risk of living with a cSFK is still a topic of debate, with kidney injury rates ranging from 6% to 60% at age 15 and limited studies in adulthood [6], [7]. In addition, large differences exist in the management of this condition. Therefore, we aim to provide practical clinical recommendations for the initial investigations, as well as indications for further diagnostics, treatment initiation, and long-term follow-up by a urologist, general practitioner, or medical specialist in children with a cSFK, based on the currently available evidence.

## 2 Evidence acquisition

We searched PubMed and EMBASE using the search strategies of previously reported systematic reviews on URA and MCDK to identify publications on cohorts of patients with a cSFK (Supplementary material) [2], [3]. Furthermore, we searched for systematic reviews (with or without a meta-analysis), randomized clinical trials, and observational studies on the different topics addressed in this article, with a preference for systematic reviews. Existing guidelines on related topics were used when appropriate. When insufficient evidence was available, recommendations were formulated in consensus meetings among the authors.

## 3 Evidence synthesis

### 3.1 Pathophysiology

Disturbances in several pathways involved in kidney development can lead to the congenital absence or reduced function of a kidney [8]. The most common causes of a cSFK are renal aplasia, URA, and unilateral MCDK, but other congenital anomalies of the kidney and urinary tract (CAKUT) may also lead to unilateral loss of kidney function [6]. As kidney development continues until the 36th week of pregnancy, a cSFK can increase in size due to both hyperplasia (ie, an increase in nephron number) and hypertrophy (ie, an increase in nephron size) [9], [10]. Hyperplasia could lead to a nephron number that is >50% of a person with two kidneys, and as such could reduce the risk of glomerular hyperfiltration and kidney injury [10], [11]. Animal models show ~50% increase in nephron numbers in the cSFK, leading to a total nephron number that equals ~70% of the total number of nephrons in an individual with two kidneys [12].

In response to a lower number of nephrons, compensatory mechanisms in the remaining nephrons result in an increase in glomerular perfusion, leading to glomerular hyperfiltration and maintenance of a stable glomerular filtration rate (GFR) [13], [14], [15]. Although beneficial in the short term, an increase in glomerular perfusion (in particular glomerular hypertension) can lead to detrimental structural changes in kidney morphology in the long term [13], [14], [15]. Following a vicious circle, glomerular hypertension leads to glomerulosclerosis with further loss of functional nephrons, which in turn increases single nephron glomerular filtration and worsens glomerular hypertension in the remaining nephrons. Glomerular hyperfiltration has been implicated as a common disease pathway shared by diabetic nephropathy, focal segmental glomerulosclerosis, SFK, and other causes of low nephron numbers, such as premature birth and low birth weight [14], [16].

Based on the hyperfiltration theory, glomerular hypertension is intermediate between low nephron endowment and progressive kidney damage. As a consequence, signs of glomerular hypertension such as albuminuria/proteinuria or systemic hypertension are expected to precede a decline in kidney function. Moreover, preventing glomerular hypertension would also prevent ongoing kidney injury [14], creating an opportunity for treatment when diagnosed early.

### 3.2 Clinical presentation

Since the introduction of structured ultrasound screening during pregnancy, an increasing number of cSFKs are detected prenatally. Antenatal diagnosis of MCDK is usually possible at the 20-wk routine ultrasound, since presentation with multiple cysts at 20 wk is rare in other diagnoses [17]. In later stages of pregnancy, an MCDK may have regressed and can be difficult to distinguish from URA [1]. Other conditions in the differential diagnosis of MCDK include severe hydronephrosis and other abdominal masses. An ectopic kidney may wrongly be diagnosed as URA on fetal ultrasound, whereas an enlarged adrenal may impose as a kidney and therefore result in missing the diagnosis of URA. Repeated antenatal ultrasound can help confirm the diagnosis and can be used to monitor the development of the unaffected kidney. In all cases, postnatal evaluation remains necessary to confirm an antenatally suspected diagnosis.

### 3.3 Assessment and diagnosis

**Fig. 1:**
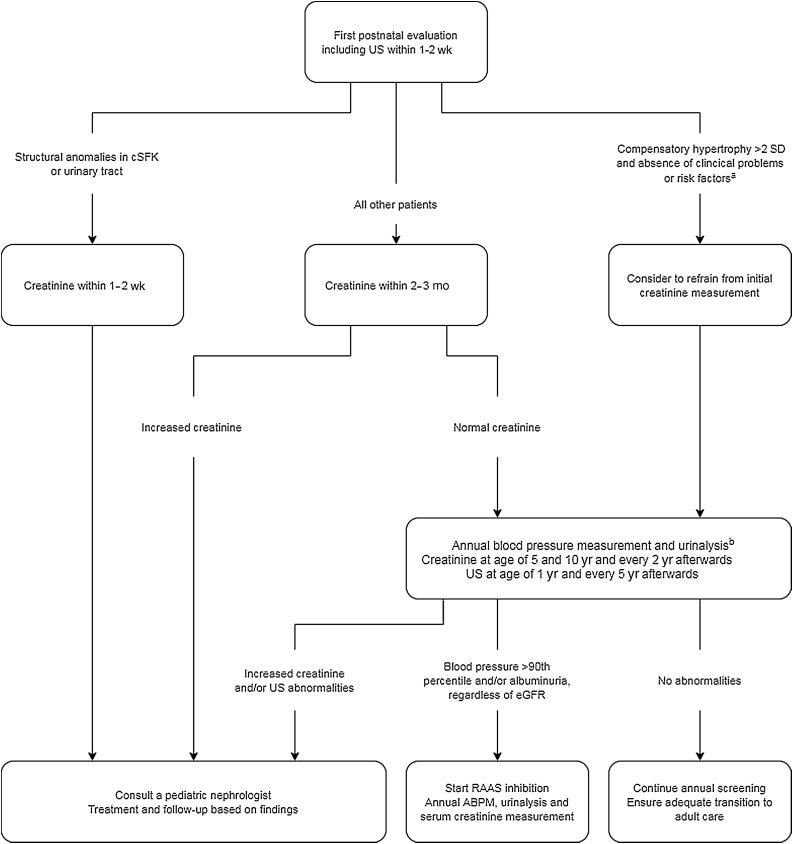
Flowchart of urological or medical management of children with a congenital solitary functioning kidney (cSFK) for whom no evidence of structural kidney anomalies is seen in the cSFK on antenatal ultrasound. ABPM = ambulatory blood pressure monitoring; eGFR = estimated glomerular filtration rate; RAAS = renin angiotensin aldosterone system; SD = standard deviation; US = kidney ultrasound. ^a^ Clinical problems or risk factors were defined as urinary tract infection, preterm birth <36 wk, dysmaturity < p10, or low birth weight (<2500 g). ^b^ A first screening can take place after approximately 3 mo, with yearly follow-up afterwards.

All children with an antenatally suspected cSFK should be referred to a pediatric urologist, pediatrician, pediatric nephrologist, or urologist depending on the local and national referral patterns for postnatal evaluation of the kidneys and urinary tract. The timing of evaluation depends on the prenatal findings; in case of suspected anomalies in the remaining kidney, early postnatal evaluation is indicated (Fig. 1).

#### 3.3.1 Ultrasound

Ultrasound screening of the kidneys and urinary tract is the main diagnostic tool for evaluation of a patient with a cSFK, given its noninvasive nature and high accuracy for diagnosing a cSFK (Table 1) [18], [19], [20]. At the first postnatal ultrasound, an attempt should be made to confirm prenatal findings and establish a definitive diagnosis. In addition, the presence of early compensatory hypertrophy with a kidney length of >2 standard deviations (SDs) above the reference value for age could identify patients with a more favorable prognosis, although follow-up studies are needed to determine the clinical significance of this finding [11]. The status of the remaining kidney and urinary tract is also highly important for the prognosis. Approximately one in three children with an cSFK have additional urogenital anomalies, including vesicoureteral reflux (VUR) in ~20% and ureteropelvic junction obstruction (UPJO) in ~5% of patients (Supplementary Table 3) [2], [3]. When such additional anomalies are found, urological advice should be sought to discuss diagnostic and treatment options.

**Table 1:** Diagnostic tools for patients with a congenital solitary functioning kidney

Modality	Advantages	Disadvantages	When indicated	When to consider
Kidney and bladder ultrasound	Noninvasive, cheap, widely available, high sensitivity and specificity for cSFK diagnosis	(Low grade) VUR or UPJO may be missed, sensitivity lower in early postnatal period and other periods of dehydration	Within 1–2 wks after birth, at 1-yr follow-up, in case of UTI	At 5, 10, and 15 yr of follow-up (especially when compensatory hypertrophy has not been shown)
Voiding cystourethrogram	Gold standard for VUR	Need for catheterization, risk of UTI, exposure to radiation	Dilated ureter on ultrasound, UTI	
MAG-3 renography	Simultaneous visualization of split kidney function and excretion	Requires intravenous injection, ectopic kidney tissue behind bladder may be missed	Suspected UPJO (high-grade hydronephrosis without VUR)	
DMSA scintigraphy	Detection of focal parenchymal abnormalities (kidney scars), split kidney function, and ectopic kidney tissue	Requires intravenous injection, time consuming	Suspected ectopic kidney	Suspected kidney scarring after pyelonephritis
Magnetic resonance urography	Detailed anatomic information, functional information can be obtained using gadolinium contrast	May require intravenous injection, catheterization, and sedation; time consuming and expensive	Unexplained symptoms after combinations of ultrasound, VCUG, and renography (eg, suspected ectopic ureteral implantation)	For surgical planning
Creatinine measurement to estimate GFR	Widely available, cheap	Invasive, influenced by maternal creatinine in postnatal period, late marker of kidney injury	After 1–2 wks or 3 mo (depending on ultrasound findings); every 5 yr afterward	When hypertension or proteinuria is found; anomalies of SFK on imaging
Urine albumin creatinine ratio measurement	Early marker of hyperfiltration, noninvasive, cheap, widely available	Risk of contamination, may be difficult to obtain in young children	Yearly follow-up visit	
Genetic screening (whole exome sequencing with kidney gene panel)	More specific diagnosis, risk of recurrence in next pregnancy of parents	Risk of incidental findings, low yield, not always available	Multiple associated anomalies	Strong positive family history, parental wish for pregnancy counseling in future
Office blood pressure measurement	Screening for hypertension, readily available	May be difficult in young children, risk of masked or white coat hypertension	Yearly in all children with cSFK	
Ambulatory blood pressure measurement	Identification of masked and white coat hypertension	Burdensome, no reference values for children <120 cm, not always available	Yearly in cSFK patients with a history of or current hypertension or CKD	All other cSFK patients

CKD = chronic kidney disease; cSFK = congenital solitary functioning kidney; DMSA = dimercaptosuccinic acid; GFR = glomerular filtration rate; MAG-3 = mercapto acetyl tri glycine; SFK = solitary functioning kidney; UPJO = ureteropelvic junction obstruction; UTI = urinary tract infection; VCUG = voiding cystourethrogram; VUR = vesicoureteral reflux.

#### 3.3.2 Voiding cystourethrogram

A voiding cystourethrogram (VCUG) is the most sensitive way to detect VUR and has frequently been used in cSFK patients given the high rate of VUR. However, as indicated in the latest guidelines for children with urinary tract infection (UTI), a high risk of VUR alone is not a proper indication for an invasive procedure such as a VCUG [21], [22]. Patients with a cSFK could be considered as having an extra indication for a VCUG as high-grade VUR appears to be a risk factor for kidney scarring [23], [24], [25], and kidney scarring can be considered to pose an additional risk in patients with an already reduced kidney mass. However, abnormalities on ultrasound are a major predictor of kidney scarring [25], and the sensitivity of kidney ultrasound to detect high-grade VUR is relatively high (60–100%) [26], [27]. Furthermore, the number of cSFK patients who need to undergo a VCUG for the diagnosis of one patient with dilating VUR is 14 and increases to 43 considering only patients who underwent ureteral reimplantation [28]. Since ultrasound is also a cheaper and less invasive approach than a VCUG, we recommend performing ultrasound as the first screening method in cSFK patients. When high-grade VUR is suspected on ultrasound or UTIs occur, we suggest the use of a VCUG as a second-line investigation to help decide whether continuous antibiotic prophylaxis or surgical correction is indicated.

#### 3.3.3 Scintigraphy

Kidney scintigraphy using radioactively labeled dimercaptosuccinic acid (DMSA) or mercapto acetyl tri glycine (MAG-3) can be used to visualize functioning kidney tissue. These studies are not needed routinely when URA or MCDK is suspected, since these diagnoses can be made accurately using sonographic studies of the kidney in >95% of cases [18], [19], [20]. Although a DMSA scan is more time consuming for the patients and parents involved, it may be indicated to visualize kidney scarring after a pyelonephritis. When ectopic kidney tissue is suspected, a DMSA scan is also indicated and preferred over a MAG-3 scan, since early bladder filling in combination with reduced/slow uptake of an ectopic kidney may result in missed ectopic kidney tissue in the bladder region using MAG-3 scintigraphy [29]. A MAG-3 scan is advised in case of significant urinary tract dilatation to exclude obstructions such as UPJO.

#### 3.3.4 Magnetic resonance imaging

Magnetic resonance imaging of the kidney and urinary tract (MRU) is a technique that can provide detailed anatomical information. When used with gadolinium as a contrast agent, functional information such as differential kidney function can be obtained simultaneously. Disadvantages of MRU include the need to lie still for a considerable amount of time and its considerable costs [30]. Furthermore, the use of a bladder catheter and intravenous administration of contrast agents may be needed, and questions about gadolinium retention in the body have not yet been answered [30]. Current use of MRU is mostly limited to patients with unexplained symptoms after extensive imaging or when detailed anatomical information is needed, for instance, for surgical planning, and in all instances the potential harm and benefit should be weighted. Use of gadolinium-based contrast agents is mainly guided by the kidney function, and there is no apparent reason to withhold this from cSFK patients for other reasons [31]. The indications for MRU may be expanded in the future, especially if its potential to assess inflammation and fibrosis or count nephron number is confirmed [30], [32], [33].

#### 3.3.5 Laboratory measurements

To confirm adequate function of the cSFK, initial screening of GFR, blood pressure, and albuminuria/proteinuria is recommended. In patients with anomalies of the cSFK on the prenatal or first postnatal ultrasound, we recommend a first serum creatinine measurement within 1–2 wks. The exact timing is a balance between the estimated reduction of kidney function, which may necessitate early evaluation, and the postnatal functional development of the kidneys, for which measurement may be postponed to the 2nd week of life. In patients without anomalies of the cSFK, creatinine measurement can take place after 2–3 mo.

Preliminary analyses of >100 cSFK patients from our own cohort show that none of the 46 children with a kidney size of >2 SDs above the mean for age (for an individual with two kidneys) have a reduced kidney function within the first year of life (unpublished data). Therefore, in absence of additional indications (clinical problems, signs of obstructive uropathy, urinary tract infections, preterm birth [<36 wk], or low birth weight [<2500 g or <p10 for gestational age]), it seems reasonable to refrain from an initial creatinine measurement in cSFK patients with compensatory hypertrophy.

#### 3.3.6 Genetic screening

With advancing knowledge on the genetic etiology of a cSFK and decreasing costs for next-generation sequencing, these techniques became available for more widespread diagnostic use. Currently, targeted sequencing studies for CAKUT seems the best option to balance the possible advantage of obtaining a specific diagnosis, such as HNF1β-related nephropathy, with the small risk of incidental findings [34]. Since screening children with a sporadic cSFK has a success rate of 10–20% [35], we currently suggest limiting genetic screening to children with additional anomalies or a positive family history.

#### 3.3.7 Screening for Müllerian anomalies

Owing to the embryological relatedness of the paramesonephric (Müllerian) and mesonephric (Wolffian) ducts, children with a cSFK often show associated anomalies of the reproductive organs [3], [36]. Since Müllerian duct anomalies can have severe and preventable complications, such as endometriosis, ultrasound screening of the internal genital organs is indicated in girls with a cSFK [37], [38]. This is possible within the first months of life due to stimulation by maternal estrogen. When a cSFK is detected in a girl, parents should be informed about the possibility of co-occurring Müllerian duct anomalies, particularly obstructed hemivagina and ipsilateral renal anomaly (OHVIRA) syndrome. After the onset of breast development, physicians should ask about menarche and cyclic abdominal pain during follow-up visits. In case of severe abdominal pain after menarche or when menarche is expected, OHVIRA and Mayer-Rokitansky-Küster-Hauser syndromes should be excluded by ultrasound.

### 3.4 Treatment and prognosis

#### 3.4.1 Antibiotic prophylaxis

In some centers, antibiotic prophylaxis was administered to cSFK patients based on the assumption that it would reduce the number of UTIs and thereby kidney scarring, especially in children with VUR or a dilated urinary tract on imaging. However, there is no evidence that administering antibiotic prophylaxis to children with a cSFK without VUR or UTIs has clinical benefits [39]. Therefore, there seems to be little ground to prescribe antibiotic prophylaxis to all children with a cSFK.

Although antibiotic prophylaxis reduces the risk of UTIs in children with VUR, a statistically significant reduction in the number of kidney scars on DMSA has not been shown [40], [41], [42]. Based on these observations, the current American Academy of Pediatrics (AAP) and National Institute for Health and Care Excellence guidelines do not recommend routine administration of antibiotic prophylaxis following a first UTI [21], [22]. However, children with a cSFK were often excluded from studies on the effects of antibiotic prophylaxis and the long-term effects of UTI [42]. We recommend a precautious approach for cSFK patients, in order to prevent scarring as an additional loss of nephrons and/or to limit the additional risk of hypertension. Therefore, we suggest administering antibiotic prophylaxis and performing a VCUG in a cSFK patient with a dilated ureter on ultrasound or after a first UTI. In addition, constipation and dysfunctional voiding should be addressed promptly, fluid intake should be encouraged, and clean toilets should be made available [21], [22]. In case of VUR and recurrent UTIs under antibiotic prophylaxis, surgical interventions can be considered.

#### 3.4.2 Follow-up ultrasounds

During follow-up, ultrasounds of the cSFK can identify compensatory hypertrophy, which has been identified as a favorable prognostic marker [6], [11], [43], [44]. The value of repeated ultrasound is unclear, however, especially after compensatory hypertrophy has been observed. The risk of malignancy in MCDK does not seem to be elevated and is not a valid reason for ultrasound screening [45]. A reasonable approach is to perform a second kidney ultrasound at the age of 1 yr, and once every 5 yr thereafter. Especially after compensatory kidney hypertrophy of >2 SDs for age has occurred, cessation of ultrasound screening can be considered.

#### 3.4.3 Screening for hyperfiltration

AAP guidelines recommend a blood pressure measurement at every medical encounter in children with chronic kidney disease (CKD), including children with structural kidney anomalies such as cSFKs [46]. Since blood pressure measurement in neonates is often imprecise, a first measurement could be performed after 3 mo and should be repeated at least yearly afterward.

Ambulatory blood pressure monitoring (ABPM) is recommended in children with CKD due to the risk of masked hypertension [46]. Evidence for masked hypertension has also been shown in SFK-specific studies (Table 2). Lack of reference values in children <120 cm and technical difficulties limit its use in children younger than 5 yr [46]. Therefore, ABPM should be considered in all children with cSFK who are ≥5 yr of age. In cSFK patients ≥5 yr of age with a history of current hypertension or CKD, ABPM should be performed yearly.

**Table 2:** Results of ambulatory blood pressure monitoring and office blood pressure readings in published cohorts of children with a (congenital) solitary functioning kidney

Author	Year	Number of patients	Normal OBP and ABPM	Masked hypertension	White coat hypertension	ABPM confirmed hypertension
Mei-Zahav [74]	2001	18 URA	18 a (100%)	0	0	0
Seeman [75]	2006	15 URA	10 (67%)	0	4 (27%)	1 (7%)
Dursun [76]	2007	22 URA	17 a (77%)	0	0	5 (23%)
Westland [77]	2014	28 cSFK	21 (75%)	5 (18%)	0	2 (7%)
Tabel [78]	2015	49 SFK b	28 (57%)	15 (31%)	0	6 (12%)
Lubrano [79]	2017	38 cSFK	27 (73%)	0	0	11 (30%)
Zambaiti [80]	2019	50 cSFK	27 (54%)	13 (26%)	0	10 (20%)
La Scola [81]	2020	81 cSFK	47 (58%)	21 (25%)	7 (9%)	6 (7%)
Total		301	195 (65%)	54 (18%)	11 (4%)	41 (14%)

ABPM = ambulatory blood pressure monitoring; cSFK = congenital solitary functioning kidney; OBP = office blood pressure; SFK = solitary functioning kidney; URA = unilateral renal agenesis.

a Results of OBP were not reported separately.

b Five children with acquired SFK included.

Based on the ESCAPE trial, the target blood pressure for children with CKD is a 24-h mean arterial pressure (MAP) of <50th percentile, which was associated with a lower risk of kidney function decline [46], [47]. In children with hypertension without CKD, the treatment goal is prevention of end organ damage, for which the target blood pressure is <90th percentile [48], [49]. Since the risk of kidney function decline is most relevant for children with a cSFK, we recommend a target blood pressure between the 50th and the 75th percentile and, if tolerated, of <50th percentile on 24-h MAP (Table 3). Reference values for blood pressure in children are provided in the latest AAP guidelines on hypertension [46].

**Table 3:** Indications for treatment with renin-angiotensin aldosterone system inhibitors

	When indicated	When to consider	Target	Comment
Blood pressure (office or ABPM)	Repeated blood pressure >90th percentile for height and gender		<50th percentile for height and gender (if tolerated); <75th percentile otherwise	Perform ABPM when office blood pressure is elevated to rule out white coat hypertension
Urine albumin creatinine ratio	>300 mg/g in first morning or 24-h urine sample	30–299 mg/g in first morning or 24-h urine sample	<30 mg/g in first morning or 24-h urine sample	
Estimated glomerular filtration rate	No data available	No data available	>90 ml/min/1.73 m^2^	Consult pediatric nephrologist when eGFR decreases >5 ml/min/1.73 m^2^ over 2 yr, or to <90 ml/min/1.73 m^2^

ABPM = ambulatory blood pressure monitoring; eGFR = estimated glomerular filtration rate.

Besides hypertension, albuminuria is another early marker of glomerular hyperfiltration [13]. Thus, urinalysis should also be performed during yearly screening visits. In healthy children, measurement of urinary albumin-to-creatinine (UAC) ratio from a first morning sample was more reliable than that from a random sample and better reflected results from 24-h urine samples [50], [51]. Since 24-h urine collection is cumbersome, especially in young children, a first morning void UAC ratio seems to be the best screening tool. In line with guidelines for patients with diabetes and those with CKD, treatment should be considered in case of modestly elevated UAC ratios (30–299 mg/g) and is strongly advised in case of a UAC ratio of ≥300 mg/g.

Since a decrease in kidney function is expected later in the course of hyperfiltration injury, serum creatinine measurement can be performed less frequently than screening for hypertension and albuminuria. Indeed, cSFK cohorts published to date showed that an isolated decrease in estimated GFR (eGFR) occurred in only 0.3–8% of their population and may especially occur during puberty [6], [7], [52], [53], [54]. Therefore, it seems reasonable to monitor eGFR once every 5 yr until the onset of puberty and every 2 yr thereafter. Even though the combination of creatinine with cystatin C provides the most accurate estimation of GFR [55], due to the limited availability of cystatin C and its higher costs, GFR can be estimated with the use of serum creatinine values in daily practice. When the eGFR decreases, a more precise estimate combining creatinine and cystatin C can be obtained, and referral to a pediatric nephrologist is indicated [55].

#### 3.4.4 Medication use

Hypertension and proteinuria are treatable risk factors for kidney function decline in children [47], [56], [57]. Although calcium-channel blockers and renin angiotensin aldosterone system (RAAS) inhibitors show similar reduction in blood pressure, the combined antihypertensive and antiproteinuric properties of RAAS inhibitors make them the recommended first-line treatment in children with a cSFK and hypertension or albuminuria [46], [58]. A decreased eGFR should not be a reason to withhold or discontinue RAAS inhibitors prescribed for hypertension and/or albuminuria. Recent data in children showed an accelerated decline of eGFR after discontinuation of RAAS inhibitors as well as an increase in albuminuria and blood pressure, suggesting that stopping RAAS inhibition might accelerate progression to kidney failure [59].

Combined use of RAAS inhibitors and nonsteroidal anti-inflammatory drugs (NSAIDs) should be avoided. In general, we recommend the use of alternatives such as acetaminophen instead of NSAIDs and refraining from NSAIDs in patients with an eGFR of <60 ml/min/1.73 m^2^ (prolonged use) or <30 ml/min/1.73 m^2^ (any use) [60]. Gentamicin use should be weighted carefully and serum levels should be monitored [60]. If possible, use of other potentially nephrotoxic drugs should also be minimized, and careful monitoring of kidney function and/or drug levels may be needed.

#### 3.4.5 Duration of follow-up

In analogy to other hyperfiltration-related kidney problems, such as diabetic nephropathy, long-term follow-up is crucial and no endpoint for follow-up can be given based on scientific evidence. Moreover, epidemiological studies have shown that the higher risk of kidney injury in patients with an SFK persists in adulthood [4]. There are even periods later in life in which stricter follow-up is needed than in childhood. Data from the ItalKid project indicate that puberty is a period with a higher risk of onset or progression of kidney injury [54]. After puberty, transition to adult care is important and efforts should be made to ensure that follow-up is continued. Since the risk of gestational hypertension and preeclampsia was 2.5-fold higher in women living with an SFK due to URA or after donor nephrectomy, pregnancy is another time when vigilance is needed [61], [62].

#### 3.4.6 Lifestyle

As children with a cSFK are at an increased risk of hypertension and kidney injury, maintaining a healthy lifestyle is of great importance. Key aspects are the avoidance of excess salt intake and obesity [63], [64], [65], [66]. Protein restriction slowed kidney function deterioration in animal models of SFKs and adults with CKD [67], [68]. However, a meta-analysis of randomized control trials testing a low protein diet in children with CKD failed to show benefits over a normal diet [69]. Children may need high protein intake to meet the demands for development and growth, and more research into optimal protein intake in children with a cSFK and/or CKD is needed before recommendations can be given.

Physical activity is an important part of a healthy lifestyle for both healthy children and children with chronic conditions. Participation in contact sports by children with a cSFK was long discouraged in fear of trauma to the remaining kidney. However, kidney injury during sports participation is extremely rare, with only nine cases per million athletic activities for American Football and even fewer in other sports [70]. Furthermore, none of these injuries resulted in kidney loss [70], [71]. Based on these data, it seems clear that the benefits of physical activity outweigh the low risk of severe kidney injury and participation by children with a cSFK should be encouraged.

### 3.5 Future perspectives

Urologists are increasingly asked to weigh the costs of their actions (for both the individual patient and the society) against the potential benefits. Since studies have shown large variation in kidney injury rates in children with cSFK, it is likely that subgroups of higher- and lower-risk children exist. Identification of these subgroups would allow for more tailored strategies to be used, and thus for a better cost-to-benefit ratio [43]. In addition, it would create an opportunity to select high-risk patients for future trials of new therapies. Performing trials in these children would be more efficient, more ethical, and more cost effective. Potential strategies that have been explored to identify the high- and low-risk subgroups are by using biomarkers, by counting nephrons in vivo using magnetic resonance imaging, and by combining already available clinical information in a prediction model [43], [72], [73]. However, all these methods need further research to be useful in clinical practice.

## 4 Conclusions

This overview points to the urological and medical clinical aspects and long-term care guidance for children with cSFKs, who are at risk of kidney injury based on glomerular hyperfiltration. After initial confirmation of the diagnosis, mainly using ultrasound, caregivers should focus on early identification of kidney injury. A yearly follow-up with checks on blood pressure and albuminuria is important, and ABPM can be a useful tool to detect masked hypertension. Estimation of the GFR should take place once every 5 yr until puberty and every 2 yr thereafter. When detected, kidney injury should be treated with RAAS inhibition and strict blood pressure control should be targeted. Since the risk of kidney injury seems increased during puberty and in pregnancy, extra checks are needed in these time periods. Adequate transition to adult care should result in continued screening in adulthood.

  ***Author contributions*:** Sander Groen in’t Woud had full access to all the data in the study and takes responsibility for the integrity of the data and the accuracy of the data analysis.

  *Study concept and design*: Groen in’t Woud, van der Zanden, Schreuder.

*Acquisition of data*: Groen in’t Woud.

*Analysis and interpretation of data*: Groen in’t Woud, Westland, Feitz, van Wijk, van der Zanden, Schreuder.

*Drafting of the manuscript:* Groen in’t Woud.

*Critical revision of the manuscript for important intellectual content:* Westland, Feitz, Roeleveld, van Wijk, van der Zanden, Schreuder.

*Statistical analysis:* None.

*Obtaining funding:* None.

*Administrative, technical, or material support:* None.

*Supervision*: Feitz, Roeleveld.

*Other*: None.

  ***Financial disclosures:*** Sander Groen in’t Woud certifies that all conflicts of interest, including specific financial interests and relationships and affiliations relevant to the subject matter or materials discussed in the manuscript (eg, employment/affiliation, grants or funding, consultancies, honoraria, stock ownership or options, expert testimony, royalties, or patents filed, received, or pending), are the following: None.

  ***Funding/Support and role of the sponsor:*** None.

  ***Acknowledgments*:**We would like to thank Dr. Kirsten Kluivers for her valuable contributions regarding the screening for abnormalities of the female reproductive tract.
