# Supplementary material for: Clinical Management of Children with a Congenital Solitary Functioning Kidney: Overview and Recommendations
Source: Eur Urol Open Sci. 2021 Feb 3;25:11–20. doi: 10.1016/j.euros.2021.01.003 (PMC8317823; doi:10.1016/j.euros.2021.01.003)
Supplement: Supplementary file 1 [file mmc1.docx]

**Online only supplements**

**Table of contents**

[**Supplementary Methods** 2](#_Toc53991303)

[Search strategy for studies with cohorts of patients with unilateral renal agenesis adopted from Westland et al^1^. 2](#_Toc53991304)

[Search strategy for studies with cohorts of patients with multicystic dysplastic kidney adopted from Schreuder et al.^2^ 2](#_Toc53991305)

[**Supplementary Table 1a:** Prevalence of unilateral renal agenesis in studies published after the previous meta-analysis was performed 3](#_Toc53991306)

[**Supplementary Table 1b:** Prevalence of multicystic dysplastic kidney in studies published after the previous meta-analysis was performed 4](#_Toc53991307)

[**Supplementary Table 2a:** Characteristics of patients with unilateral renal agenesis from published cohorts 5](#_Toc53991308)

[**Supplementary Table 2b:** Characteristics of patients with multicystic dysplastic kidney from published cohorts 6](#_Toc53991309)

[**Supplementary Table 3a:** Prevalence of associated urinary tract anomalies in children with unilateral renal agenesis from published cohorts 8](#_Toc53991310)

[**Supplementary Table 3b:** Prevalence of associated urinary tract anomalies in children with multicystic dysplastic kidney from published cohorts 9](#_Toc53991311)

[**References** 10](#_Toc53991312)

# **Supplementary Methods**

## Search strategy for studies with cohorts of patients with unilateral renal agenesis adopted from Westland et al^1^.

Pubmed search:

| **Search** | **Query** |
| --- | --- |
| **#5** | #4 Filters: Publication date from 2012/01/01 |
| **#4** | #3 NOT (animals [mh] NOT humans [mh]) |
| **#3** | #1 NOT #2 |
| **#2** | "Case Reports" [Publication Type] |
| **#1** | "Hereditary renal agenesis" [Supplementary Concept] OR single kidney*[tiab] OR absent kidney*[tiab] OR solitary functioning kidney*[tiab] OR solitary kidney*[tiab] OR unilateral renal agenesis[tiab] OR hereditary renal agenesis[tiab] OR unilateral renal aplasia[tiab] OR hereditary renal aplasia[tiab] OR aplastic kidney*[tiab] |

OVID search:

| **Search** | **Query** |
| --- | --- |
| **#6** | 5 Filters: Publication date from 2012/01/01 |
| **#5** | #4 NOT #3 |
| **#4** | #1 NOT #2 |
| **#3** | 'animal'/exp OR 'animal experiment'/exp NOT 'human'/exp |
| **#2** | 'case report'/exp |
| **#1** | exp 'solitary kidney'/ or (single adj3 kidney*).ab,ti. or (absent adj3 kidney*).ab,ti. or ('solitary functioning' adj2 kidney*).ab,ti. or ('unilateral renal' adj2 agenesis).ab,ti. or ('hereditary renal' adj2 agenesis).ab,ti. or ('unilateral renal' adj2 aplasia).ab,ti. or ('hereditary renal' adj2 aplasia).ab,ti. or (aplastic adj3 kidney*).ab,ti. |

## Search strategy for studies with cohorts of patients with multicystic dysplastic kidney adopted from Schreuder et al.^2^

Pubmed search:

| **Search** | **Query** |
| --- | --- |
| **#2** | Filters: Publication date from 2008/01/01 |
| **#1** | ((("multicystic dysplastic kidney") OR ("multicystic kidney dysplasia")) OR "Multicystic Dysplastic Kidney"[Mesh]) |

EMBASE search:

| **Search** | **Query** |
| --- | --- |
| **#2** | Filters: Publication date from 2008/01/01 |
| **#1** | ‘multicystic’ AND ‘dysplastic’ or ‘dysplasia’ AND ‘kidney’ |

# **Supplementary Table 1a:** Prevalence of unilateral renal agenesis in studies published after the previous meta-analysis was performed

| **Author** | **Year** | **Country** | **Age at diagnosis** | **Patients with URA** | **Size screened population** | **Prevalence** |
| --- | --- | --- | --- | --- | --- | --- |
| Laurichesse Delmas^3^ | 2017 | France | Prenatal with postnatal confirmation | 177 | 447 885 | 1 : 2 530 |
| Bakker^4^ | 2018 | Netherlands | Prenatal with postnatal confirmation | 47 | 119 297 | 1 : 2 538 |
| Li^5^ | 2019 | China | Prenatal with postnatal confirmation | 529 | 1 748 038 | 1 : 3 304 |
| Syngelaki^6, a^ | 2019 | UK | Prenatal with postnatal confirmation | 124 | 100 997 | 1 : 814 |
| Zmora^7^ | 2019 | Israel | Prenatal with postnatal confirmation | 49 | 59 382 | 1 : 1 211 |
| **Total** |  |  |  | **926** | **2 475 599** | **1 : 2 673** |
| Westland^1^ | 2013 | Meta-analysis |  | 2 094 | 4 253 483 | 1 : 2 031 |
| **Total including previous meta-analysis** | | | | **3 020** | **6 729 082** | **1 : 2 228** |

URA unilateral renal agenesis; UK United Kingdom

^a^ Included patients empty renal fossa without differentiation between unilateral renal agenesis and ectopic kidney.

# **Supplementary Table 1b:** Prevalence of multicystic dysplastic kidney in studies published after the previous meta-analysis was performed

| **Author** | **Year** | **Country** | **Age at diagnosis** | **Patients with MCDK** | **Size screened population** | **Prevalence** |
| --- | --- | --- | --- | --- | --- | --- |
| Saha^8^ | 2008 | India | Prenatal with postnatal confirmation | 3 | 6 682 | 1 : 2 227 |
| Cordero^9^ | 2009 | USA | Prenatal with postnatal confirmation | 19 | 15 000 | 1 : 789 |
| Halek^10^ | 2010 | Czech Republic | Neonatal screening | 4 | 6 088 | 1 : 1 522 |
| Melo^11^ | 2012 | Brazil | Neonatal screening | 35 | 29 653 | 1 : 847 |
| Al Naimi^12^ | 2013 | Germany | Prenatal | 33 | 16 000 | 1 : 485 |
| Beke^13^ | 2014 | Hungary | Pre- and early postnatal | 17 | 19 602 | 1 : 1 153 |
| Bondagji^14^ | 2014 | Saudi Arabia | Prenatal with postnatal confirmation | 13 | 43 209 | 1 : 3 324 |
| Winding^15^ | 2014 | Europe | Pre- and early postnatal | 391 | 1 458 552 | 1 : 3 730 |
| Gong^16^ | 2018 | China | Neonatal screening | 1 | 8 827 | 1 : 8 827 |
| Chen^17^ | 2019 | Taiwan | Pre- and postnatal | 298 | 2 033 004 | 1 : 6 822 |
| **Total** |  |  |  | **814** | **3 636 617** | **1 : 4 467** |
| Schreuder^2^ | 2009 | Meta-analysis |  | 371 | 1 588 271 | 1 : 4 281 |
| **Total including previous meta-analysis^a^** | | | | **1 080** | **4 515 858** | **1 : 4 181** |

MCDK multicystic dysplastic kidney; USA United States of America

^a^ The results from Wiesel *et al^18^ (105 cases among 709 030 births)* were removed from the original meta-analysis because of overlap with Winding et al.

# **Supplementary Table 2a:** Characteristics of patients with unilateral renal agenesis from published cohorts

| **Author** | **Year** | **Number of patients** | **Left-sided URA**  **N (%)** | **Male**  **N (%)** |
| --- | --- | --- | --- | --- |
| Castellano-Martinez^19^ | 2016 | 21 | 12 (57%) | 9 (43%) |
| Clinton^20, a^ | 2016 | 36 | 18 (50%) | NR |
| Davidovits^21^ | 2017 | 20 | NR | 13 (65%) |
| Dogan^22^ | 2013 | 51 | 33 (65%) | 31 (61%) |
| Kostadinova^23^ | 2016 | 10 | NR | 6 (60%) |
| Laurichesse Delmas^3^ | 2017 | 177 | 101 (57%) | NR |
| La Scola^24, a^ | 2016 | 42 | 24 (57%) | NR |
| La Scola^25, a^ | 2020 | 18 | 8 (44%) | NR |
| Li^5^ | 2019 | 529 | NR | 241 (46%) |
| Marzuillo^26, a^ | 2017 | 199 | 99 (50%) | NR |
| Perlman^27^ | 2016 | 74 | 30 (41%) | NR |
| Sarhan^28^ | 2016 | 46 | 12 (26%) | 24 (52%) |
| Stefanowicz^29^ | 2012 | 17 | NR | 8 (47%) |
| Xu^30^ | 2019 | 118 | 59 (50%) | 62 (53%) |
| Zmora^7^ | 2019 | 49 | 23 (47%) | 37 (76%) |
| **Total**^b^ |  | 1 407 | 419 (51%) | 431 (52%) |
| Westland^1^ | 2013 | 2 684 | 1 080 (52%) | 1 542 (63%) |
| **Updated total**^b^ |  | 4 091 | 1 499 (52%) | 1 973 (60%) |

URA unilateral renal agenesis; NR not reported

^a^ Additional information obtained through personal communication with the authors.

^b^ Percentage reported as the number of patients with the characteristic divided by the number of patients for which the characteristic was reported.

# **Supplementary Table 2b:** Characteristics of patients with multicystic dysplastic kidney from published cohorts

| **Author** | **Year** | **Number of patients** | **Left-sided MCDK**  **N (%)** | **Male**  **N (%)** |
| --- | --- | --- | --- | --- |
| Akil^31^ | 2012 | 33 | NR | 17 (52%) |
| Al Naimi^12^ | 2013 | 33 | 14 (42%) | 22 (67%) |
| Alsaif^32^ | 2019 | 44 | 22 (50%) | 25 (57%) |
| Ayaz^33^ | 2017 | 21 | 10 (48%) | NR |
| Aytac^34^ | 2011 | 20 | 12 (60%) | 18 (90%) |
| Balasundaram^35^ | 2018 | 38 | 20 (52%) | 22 (58%) |
| Bartoli^36^ | 2018 | 14 | NR | 4 (29%) |
| Blachman-Braun^37^ | 2020 | 156 | 83 (53%) | 98 (63%) |
| Brown^38^ | 2019 | 3 792 | NR | 2 072 (55%) |
| Brown^39^ | 2019 | 165 | 93 (56%) | 82 (50%) |
| Carazo-Palacias^40^ | 2016 | 56 | 33 (59%) | 38 (68%) |
| Chijioke^41^ | 2010 | 6 | 1 (17%) | NR |
| Clinton^20, a^ | 2016 | 28 | 15 (54%) | NR |
| Davidovits^21^ | 2017 | 12 | NR | 8 (67%) |
| Dogan^42^ | 2014 | 59 | 26 (44%) | 31 (53%) |
| Eickmeyer^43^ | 2014 | 301 | 141 (47%) | 165 (55%) |
| Faruque^44^ | 2020 | 106 | 55 (52%) | 63 (59%) |
| Gaither^45^ | 2018 | 443 | 238 (54%) | 235 (53%) |
| Gokce^46^ | 2012 | 25 | 12 (48%) | NR |
| Halek^10^ | 2010 | 4 | NR | 1 (25%) |
| Hayes^47^ | 2012 | 323 | 166 (51%) | 182 (56%) |
| Hsu^48^ | 2012 | 34 | 15 (44%) | NR |
| Kara^49^ | 2018 | 128 | 66 (52%) | 82 (64%) |
| Kashiwagi^50^ | 2018 | 4 | 2 (50%) | 1 (25%) |
| Kiyak^51^ | 2009 | 90 | 46 (51%) | 53 (59%) |
| Kumar^52^ | 2019 | 51 | 17 (33%) | 29 (57%) |
| La Scola^24, a^ | 2016 | 55 | 33 (60%) | NR |
| La Scola^25, a^ | 2020 | 19 | 11 (58%) | NR |
| Mansoor^53^ | 2011 | 121 | 65 (54%) | 60 (50%) |
| Marzuillo^54, a^ | 2017 | 185 | 107 (58%) | NR |
| Mashat^55^ | 2015 | 16 | NR | 12 (75%) |
| Matsumura^56^ | 2018 | 17 | 7 (41%) | 7 (41%) |
| Mattioli^57^ | 2010 | 12 | NR | 8 (67%) |
| Moralioglu^58^ | 2014 | 68 | 35 (51%) | 40 (59%) |
| Poggiali^59^ | 2019 | 132 | NR | 74 (56%) |
| Sanna-Cherchi^60^ | 2009 | 40 | NR | 19 (48%) |
| Sarhan^61^ | 2014 | 63 | 32 (51%) | 35 (56%) |
| Scala^62b^ | 2017 | 94 | 70 (50%) | NR |
| Sharada^63^ | 2014 | 47 | 22 (47%) | 27 (57%) |
| Singh^64^ | 2009 | 22 | 12 (55%) | 18 (82%) |
| Soliman^65^ | 2015 | 17 | NR | 10 (59%) |
| Tiryaki^66,b^ | 2013 | 102 | 18 (39%) | 61 (60%) |
| van Vuuren^67^ | 2012 | 60 | 27 (45%) | NR |
| Weinstein^68^ | 2008 | 80 | 46 (58%) | 42 (53%) |
| Westland^69^ | 2013 | 124 | 61 (49%) | 81 (65%) |
| Whittam^70^ | 2014 | 91 | 54 (59%) | 57 (63%) |
| Yamamoto^71^ | 2019 | 75 | 35 (47%) | 29 (39%) |
| Zambaiti^72^ | 2019 | 40 | 19 (48%) | 27 (68%) |
| **Total**^b^ |  | 7 466 | 1 741 (51%) | 3 855 (56%) |
| Schreuder^2^ | 2009 | 3 557 | 1 663 (53%) | 1 791 (59%) |
| **Updated total**^b^ |  | 11 022 | 3 404 (52%) | 5 646 (57%) |

MCDK multicystic dysplastic kidney; NR not reported

a Additional information obtained through personal communication with the authors.

b Percentage reported as the number of patients with the characteristic divided by the number of patients for which the characteristic was reported.

# **Supplementary Table 3a:** Prevalence of associated urinary tract anomalies in children with unilateral renal agenesis from published cohorts

| **Author** | **Year** | **Number of patients** | **Patients with VCUG**  **N (%^a^)** | **Patients with VUR**  **N (%^b^)** | **Mild VUR (grade I-II)**  **N (%^c^)** | **Severe VUR (grade III-V)**  **N (%^c^)** | **Patients with UPJO**  **N (%^b^)** |
| --- | --- | --- | --- | --- | --- | --- | --- |
| Dogan^22^ | 2013 | 51 | 15 (29%) | 3 (20%) | NR | NR | 2 (13%) |
| Laurichesse Delmas^3^ | 2017 | 177 | NR | 5 (NR) | NR | NR | 6 (NR) |
| Sarhan^28^ | 2016 | 46 | 20 (43%) | 4 (20%) | NR | NR | NR |
| **Total** |  | 274 | 35 (36%) | 7 (20%) | NR | NR | 8 (13%) |
| Westland^1^ | 2013 | 2 684 | 770 (29%) | 184 (24%) | NR | NR | 38 (4.9%) |
| **Updated total** |  | 2 958 | 805 (29%) | 191 (24%) | NR | NR | 40 (5.0%) |

VCUG voiding cystourethrogram; VUR vesicoureteral reflux; UPJO ureteropelvic junction obstruction; NR not reported.

^a^ Calculated as percentage of total number of patients in the studies for which the number of patients who underwent VCUG was reported. ^b^ Calculated as percentage of number of patients with VCUG. ^c^ Calculated as percentage of patients with VUR.

# **Supplementary Table 3b:** Prevalence of associated urinary tract anomalies in children with multicystic dysplastic kidney from published cohorts

| **Author** | **Year** | **Number of patients** | **Patients with VCUG**  **N (%^a^)** | **Patients with VUR**  **N (%^b^)** | **Mild VUR (grade I-II)**  **N (%^c^)** | **Severe VUR (grade III-V)**  **N (%^c^)** | **Patients with UPJO**  **N (%^b^)** |
| --- | --- | --- | --- | --- | --- | --- | --- |
| Akil^31^ | 2012 | 33 | 29 (88%) | 10 (34%) | 5 (50%) | 5 (50%) | 1 (3.4%) |
| Alsaif^32^ | 2019 | 44 | 43 (98%) | 7 (16%) | 6 (86%) | 1 (14%) | 0 (0%) |
| Aytac^34^ | 2011 | 20 | 20 (100%) | 3 (15%) | NR | NR | 3 (15%) |
| Balasundaram^35^ | 2018 | 38 | 35 (92%) | 2 (5.7%) | 1 (50%) | 1 (50%) | 0 (0%) |
| Bartoli^36^ | 2018 | 14 | 14 (100%) | 2 (14%) | NR | NR | 2 (14%) |
| Blachman-Braun^37^ | 2020 | 156 | 156 (100%) | 34 (22%) | 18 (72%) | 7 (28%) | NR |
| Brown^39^ | 2019 | 165 | 77 (47%) | 18 (23%) | 7 (39%) | 11 (61%) | NR |
| Calaway^73^ | 2014 | 133 | 133 (100%) | 23 (17%) | 12 (52%) | 11 (48%) | NR |
| Calisti^74^ | 2008 | 26 | 26 (100%) | 2 (7.7%) | NR | NR | 2 (7.7%) |
| Carazo-Palacias^40^ | 2016 | 56 | NR | 8 (NR) | 5 (63%) | 3 (37%) | 3 (NR) |
| Cordero^9^ | 2009 | 19 | 5 (26%) | 2 (40%) | NR | NR | NR |
| Dogan^42^ | 2014 | 59 | 30 (51%) | 8 (27%) | 2 (25%) | 6 (75%) | 3 (10%) |
| Eickmeyer^43^ | 2014 | 301 | 239 (79%) | 53 (22%) | NR | NR | 10 (4.2%) |
| Faruque^44^ | 2020 | 106 | 106 (100%) | 13 (12%) | 0 (0%) | 4 (100%) | 6 (5.7%) |
| Gaither^45^ | 2018 | 443 | 183 (41%) | 20 (11%) | NR | NR | NR |
| Gokce^46^ | 2012 | 25 | 25 (100%) | 6 (24%) | NR | NR | 1 (4.4%) |
| Hayes^47^ | 2012 | 323 | NR | 37 (NR) | NR | NR | 9 (NR) |
| Kara^49^ | 2018 | 128 | 74 (58%) | 15 (20%) | 6 (67%) | 3 (33%) | 6 (8.1%) |
| Kiyak^51^ | 2009 | 90 | 90 (100%) | 7 (7.8%) | 5 (71%) | 2 (29%) | 1 (1.1%) |
| Mansoor^53^ | 2011 | 121 | 101 (83%) | 17 (17%) | 11 (65%) | 6 (35%) | 5 (5.0%) |
| Marzuillo^54^ | 2017 | 185 | NR | 8 (NR) | 3 (38%) | 5 (62%) | 1 (NR) |
| Matsumura^56^ | 2018 | 17 | NR | 1 (NR) | NR | NR | 2 (NR) |
| Mattioli^57^ | 2010 | 12 | NR | 2 (NR) | NR | NR | NR |
| Moralioglu^58^ | 2014 | 68 | 68 (100%) | 5 (7.4%) | 1 (20%) | 4 (80%) | 1 (1.5%) |
| Sarhan^61, d^ | 2014 | 63 | 63 (100%) | 20 (32%) | 14 (88%) | 2 (12%) | NR |
| Shankar^75^ | 2018 | 24 | 18 (75%) | 3 (17%) | 1 (33%) | 2 (67%) | NR |
| Sharada^63^ | 2014 | 47 | 47 (100%) | 13 (28%) | 6 (50%) | 6 (50%) | NR |
| Singh^64^ | 2009 | 22 | NR | 3 (NR) | NR | NR | 3 (NR) |
| Tiryaki^66^ | 2013 | 102 | 84 (82%) | 14 (17%) | NR | NR | 8 (9.5%) |
| Yamamoto^71^ | 2019 | 75 | 75 (100%) | 8 (11%) | 4 (67%) | 2 (33%) | NR |
| **Total** |  | 2 915 | 1 741 (60%) | 364 (21%) | 107 (57%) | 81 (43%) | 71 (4.1%) |
| Schreuder^2^ | 2009 | 3 557 | 2 104 (59%) | 415 (20%) | 100 (60%) | 68 (40%) | 103 (4.8%) |
| **Updated total** |  | 6 472 | 3 845 (59%) | 779 (20%) | 207 (58%) | 149 (42%) | 174 (4.5%) |

VCUG voiding cystourethrogram; VUR vesicoureteral reflux; UPJO ureteropelvic junction obstruction; NR not reported.

^a^ Calculated as percentage of total number of patients in the studies for which the number of patients who underwent VCUG was reported. ^b^ Calculated as percentage of number of patients with VCUG. ^c^ Calculated as percentage of patients with VUR for whom grade was reported. ^d^ Mild was classified as grade I-III, severe as grade IV or V.

# **References**

1. Westland R, Schreuder MF, Ket JC, van Wijk JA. Unilateral renal agenesis: a systematic review on associated anomalies and renal injury. *Nephrology Dialysis Transplantation.* 2013;28(7):1844-1855.

2. Schreuder MF, Westland R, van Wijk JA. Unilateral multicystic dysplastic kidney: a meta-analysis of observational studies on the incidence, associated urinary tract malformations and the contralateral kidney. *Nephrol Dial Transplant.* 2009;24(6):1810-1818.

3. Laurichesse Delmas H, Kohler M, Doray B, et al. Congenital unilateral renal agenesis: Prevalence, prenatal diagnosis, associated anomalies. Data from two birth-defect registries. *Birth defects research.* 2017;109(15):1204-1211.

4. Bakker MK, Bergman JEH, Fleurke-Rozema H, et al. Prenatal diagnosis of urinary tract anomalies, a cohort study in the Northern Netherlands. *Prenat Diagn.* 2018;38(2):130-134.

5. Li ZY, Chen YM, Qiu LQ, et al. Prevalence, types, and malformations in congenital anomalies of the kidney and urinary tract in newborns: a retrospective hospital-based study. *Ital J Pediatr.* 2019;45.

6. Syngelaki A, Hammami A, Bower S, Zidere V, Akolekar R, Nicolaides KH. Diagnosis of fetal non-chromosomal abnormalities on routine ultrasound examination at 11-13 weeks' gestation. *Ultrasound Obstet Gynecol.* 2019;54(4):468-+.

7. Zmora O, Beloosesky R, Gover A, Bronshtein M. Unilateral Renal Agenesis Diagnosed on Early Prenatal Transvaginal Scans. *Isr Med Assoc J.* 2019;21(2):85-87.

8. Saha A, Batra P, Chaturvedi P, Mehera B, Tayade A. Antenatal detection of renal malformations. *Indian Pediatr.* 2009;46(4):346-348.

9. Cordero L, Nankervis CA, Oshaughnessy RW, Koff SA, Giannone PJ. Postnatal follow-up of antenatal hydronephrosis: a health-care challenge. *J Perinatol.* 2009;29(5):382-387.

10. Halek J, Flogelova H, Michalkova K, et al. Diagnostic accuracy of postnatal ultrasound screening for urinary tract abnormalities. *Pediatr Nephrol.* 2010;25(2):281-287.

11. Melo BF, Aguiar MB, Bouzada MC, et al. Early risk factors for neonatal mortality in CAKUT: analysis of 524 affected newborns. *Pediatr Nephrol.* 2012;27(6):965-972.

12. Al Naimi A, Baumuller JE, Spahn S, Bahlmann F. Prenatal diagnosis of multicystic dysplastic kidney disease in the second trimester screening. *Prenat Diagn.* 2013;33(8):726-731.

13. Beke A, Eros FR, Pete B, Szabo I, Gorbe E, Rigo J. Efficacy of prenatal ultrasonography in diagnosing urogenital developmental anomalies in newborns. *BMC Pregnancy Childbirth.* 2014;14.

14. Bondagji NS. Antenatal diagnosis, prevalence and outcome of congenital anomalies of the kidney and urinary tract in Saudi Arabia. *Urol Ann.* 2014;6(1):36-40.

15. Winding L, Loane M, Wellesley D, et al. Prenatal diagnosis and epidemiology of multicystic kidney dysplasia in Europe. *Prenat Diagn.* 2014;34(11):1093-1098.

16. Gong Y, Zhang Y, Shen Q, et al. Early detection of congenital anomalies of the kidney and urinary tract: cross-sectional results of a community-based screening and referral study in China. *BMJ Open.* 2018;8(5).

17. Chen LJ, Chiou JY, Huang JY, Su PH, Chen JY. Birth defects in Taiwan: A 10-year nationwide population-based, cohort study. *Journal of the Formosan Medical Association.* 2020;119(1):553-559.

18. Wiesel A, Queisser-Luft A, Clementi M, Bianca S, Stoll C, Grp ES. Prenatal detection of congenital renal malformations by fetal ultrasonographic examination: An analysis of 709,030 births in 12 European countries. *Eur J Med Genet.* 2005;48(2):131-144.

19. Castellano-Martinez A, Rodriguez-Gonzalez M, Roldan-Cano V. Early kidney damage in patients born with unilateral renal agenesis. *An Pediatr.* 2017;87(3):171-173.

20. Clinton CM, Chasen ST. Unilateral Fetal Renal Abnormalities Are They Really Isolated? *J Ultrasound Med.* 2016;35(3):561-564.

21. Davidovits M, Cleper R, Eizenberg N, Hocherman O, Mashiach R. Outcomes of prenatally diagnosed solitary functioning kidney during early life. *J Perinatol.* 2017;37(12):1325-1329.

22. Dogan CS, Torun Bayram M. Renal outcome of children with unilateral renal agenesis. *Turk J Pediatr.* 2013;55(6):612-615.

23. Kostadinova ES, Miteva LD, Stanilova SA. ACE serum level and I/D gene polymorphism in children with obstructive uropathies and other congenital anomalies of the kidney and urinary tract. *Nephrology.* 2017;22(8):609-616.

24. La Scola C, Ammenti A, Puccio G, et al. Congenital Solitary Kidney in Children: Size Matters. *J Urol.* 2016;196(4):1250-1256.

25. La Scola C, Marra G, Ammenti A, et al. Born with a solitary kidney: at risk of hypertension. *Pediatric Nephrology.* 2020;35(8):1483-1490.

26. Marzuillo P, Guarino S, Grandone A, et al. Outcomes of a Cohort of Prenatally Diagnosed and Early Enrolled Patients with Congenital Solitary Functioning Kidney. *J Urol.* 2017;198(5):1153-1158.

27. Perlman S, Lotan D, Dekel B, et al. Prenatal compensatory renal growth in unilateral renal agenesis. *Prenat Diagn.* 2016;36(11):1075-1080.

28. Sarhan OM, Albedaiwi K, Al Harbi B, Al Otay A, Al Ghanbar M, Nakshabandi Z. Unilateral Renal Agenesis: Necessity of Postnatal Evaluation in a Contemporary Series. *Urology.* 2016;98:144-148.

29. Stefanowicz J, Owczuk R, Kalluzynska B, et al. Renal function and solitary kidney disease: Wilms tumour survivors versus patients with unilateral renal agenesis. *Kidney and Blood Pressure Research.* 2012;35(3):174-181.

30. Xu Q, Wu H, Zhou L, et al. The clinical characteristics of Chinese patients with unilateral renal agenesis. *Clin Exp Nephrol.* 2019;23(6):792-798.

31. Akil I, Biyikli N, Yazici P, Ozyurt BC, Alpay H. Microalbumin excretion and outcome in children with multicystic dysplastic kidney. *Turk J Med Sci.* 2012;42(6):1039-1043.

32. Alsaif A, Alsadoun F, Alsaef AM, Ahmed I, Ali K. Outcome of Infants with Antenatally Diagnosed Multicystic Dysplastic Kidney. *J Clin Neonatol.* 2019;8(1):34-38.

33. Ayaz S, Dilli A, Gultekin SS, Ayaz UY. Cyst-to-kidney volume ratio in the sonographic diagnosis of unilateral multicystic dysplastic kidney in children. *Med Ultrason.* 2017;19(2):159-165.

34. Aytac B, Sehitoglu I, Vuruskan H. Multicystic Dysplastic Kidney: Four-Year Evaluation. *Turk J Pathol.* 2011;27(3):210-214.

35. Balasundaram M, Chock VY, Wu HY, Blumenfeld YJ, Hintz SR. Predictors of poor neonatal outcomes in prenatally diagnosed multicystic dysplastic kidney disease. *J Perinatol.* 2018;38(6):658-664.

36. Bartoli F, Pastore V, Cale I, et al. Prospective Study on Several Urinary Biomarkers as Indicators of Renal Damage in Children with CAKUT. *Eur J Pediatr Surg.* 2018;04:04.

37. Blachman-Braun R, Camp MM, Becerra MF, et al. Voiding Cystourethrogram in Children With Unilateral Multicystic Dysplastic Kidney: Is It Still necessary? *Urology.* 2020.

38. Brown CT, Sebastiao YV, McLeod DJ. Trends in surgical management of multicystic dysplastic kidney at USA children's hospitals. *J Pediatr Urol.* 2019;15(4):368-373.

39. Brown C, McLeod D, Ching C. Knowledge of vesicoureteral reflux obtained by screening voiding cystourethrogram in children with multicystic dysplastic kidney does not change patient management or prevent febrile urinary tract infection. *J Pediatr Urol.* 2019;15(3).

40. Carazo-Palacios ME, Couselo-Jerez M, Serrano-Durba A, et al. Multicystic dysplastic kidney: Assessment of the need for renal scintigraphy and the safety of conservative treatment. *Actas Urologicas Espanolas.* 2017;41(1):62-67.

41. Chijioke A, Aderibigbe A, Olarenwaju TO, Makusidi AM, Oguntoyinbo AE. Prevalence and pattern of cystic kidney diseases in Ilorin, Nigeria. *Saudi J Kidney Dis Transpl.* 2010;21(6):1172-1178.

42. Dogan CS, Torun-Bayram M, Aybar MD. Unilateral multicystic dysplastic kidney in children. *Turk J Pediatr.* 2014;56(1):75-79.

43. Eickmeyer AB, Casanova NF, He C, et al. The natural history of the multicystic dysplastic kidney - Is limited follow-up warranted? *J Pediatr Urol.* 2014;10(4):655-661.

44. Faruque A, Narayanan S, Marley I, et al. Multicystic dysplastic kidney - treat each case on its merits. *Journal of Pediatric Surgery.* 2020.

45. Gaither TW, Patel A, Patel C, Chuang KW, Cohen RA, Baskin LS. Natural History of Contralateral Hypertrophy in Patients with Multicystic Dysplastic Kidneys. *J Urol.* 2018;199(1):280-286.

46. Gokce I, Biyikli N, Tugtepe H, Tarcan T, Alpay H. Clinical spectrum of antenatally detected urinary tract abnormalities with respect to hydronephrosis at postnatal ultrasound scan. *Pediatr Surg Int.* 2012;28(5):543-552.

47. Hayes WN, Watson AR. Unilateral multicystic dysplastic kidney: does initial size matter? *Pediatr Nephrol.* 2012;27(8):1335-1340.

48. Hsu PY, Yu CH, Lin K, Cheng YC, Chang CH, Chang FM. Prenatal diagnosis of fetal multicystic dysplastic kidney in the era of three-dimensional ultrasound: 10-year experience. *Taiwan J Obstet Gynecol.* 2012;51(4):596-602.

49. Kara A, Gurgoze MK, Aydin M, Koc ZP. Clinical features of children with multicystic dysplastic kidney. *Pediatr Int.* 2018;60(8):750-754.

50. Kashiwagi Y, Agata K, Go S, Nishimata S, Kawashima H. Clinical features of solitary functioning kidney. *CEN Case Reports.* 2018;7(1):185-186.

51. Kiyak A, Yilmaz A, Turhan P, Sander S, Aydin G, Aydogan G. Unilateral multicystic dysplastic kidney: single-center experience. *Pediatric Nephrology.* 2009;24(1):99-104.

52. Kumar BH, Krishnamurthy S, Chandrasekaran V, Jindal B, Ananthakrishnan R. Clinical Spectrum of Congenital Anomalies of Kidney and Urinary Tract in Children. *Indian Pediatr.* 2019;56(7):566-570.

53. Mansoor O, Chandar J, Rodriguez MM, et al. Long-term risk of chronic kidney disease in unilateral multicystic dysplastic kidney. *Pediatr Nephrol.* 2011;26(4):597-603.

54. Marzuillo P, Guarino S, Grandone A, et al. Outcomes of a Cohort of Prenatally Diagnosed and Early Enrolled Patients with Congenital Solitary Functioning Kidney. *J Urol.* 2017;198(5):1153-1158.

55. Mashat S, El Desoky S, Kari J. Outcome of multicystic dysplastic kidneys in children. *Pediatric Nephrology.* 2015;30 (9):1595.

56. Matsumura K, Sugii K, Awazu M. Trajectory of Estimated Glomerular Filtration Rate Predicts Renal Injury in Children with Multicystic Dysplastic Kidney. *Nephron.* 2018:1-6.

57. Mattioli G, Pini-Prato A, Costanzo S, et al. Nephrectomy for multicystic dysplastic kidney and renal hypodysplasia in children: where do we stand? *Pediatr Surg Int.* 2010;26(5):523-528.

58. Moralioglu S, Celayir AC, Bosnali O, Pektas OZ, Bulut IK. Single center experience in patients with unilateral multicystic dysplastic kidney. *J Pediatr Urol.* 2014;10(4):763-768.

59. Poggiali IV, Silva ACSE, Vasconcelos MA, et al. A clinical predictive model of renal injury in children with congenital solitary functioning kidney. *Pediatric Nephrology.* 2019;34(3):465-474.

60. Sanna-Cherchi S, Ravani P, Corbani V, et al. Renal outcome in patients with congenital anomalies of the kidney and urinary tract. *Kidney Int.* 2009;76(5):528-533.

61. Sarhan OM, Alghanbar M, Alsulaihim A, Alharbi B, Alotay A, Nakshabandi Z. Multicystic dysplastic kidney: Impact of imaging modality selection on the initial management and prognosis. *J Pediatr Urol.* 2014;10(4):645-649.

62. Scala C, McDonnell S, Murphy F, et al. Diagnostic accuracy of midtrimester antenatal ultrasound for multicystic dysplastic kidneys. *Ultrasound Obstet Gynecol.* 2017;50(4):464-469.

63. Sharada S, Vijayakumar M, Nageswaran P, Ekambaram S, Udani A. Multicystic dysplastic kidney: a retrospective study. *Indian Pediatr.* 2014;51(8):641-643.

64. Singh JK, Kanojia RP, Narasimhan KL. Multicystic dysplastic kidney in children - A need for conservative and long term approach. *Indian J Pediatr.* 2009;76(8):809-812.

65. Soliman NA, Ali RI, Ghobrial EE, Habib EI, Ziada AM. Pattern of clinical presentation of congenital anomalies of the kidney and urinary tract among infants and children. *Nephrology.* 2015;20(6):413-418.

66. Tiryaki S, Alkac AY, Serdaroglu E, Bak M, Avanoglu A, Ulman I. Involution of multicystic dysplastic kidney: is it predictable? *J Pediatr Urol.* 2013;9(3):344-347.

67. van Vuuren SH, Sol CM, Broekhuizen R, et al. Compensatory Growth of Congenital Solitary Kidneys in Pigs Reflects Increased Nephron Numbers Rather Than Hypertrophy. *PLoS One.* 2012;7 (11) (no pagination)(e49735).

68. Weinstein A, Goodman TR, Iragorri S. Simple multicystic dysplastic kidney disease: End points for subspecialty follow-up. *Pediatric Nephrology.* 2008;23(1):111-116.

69. Westland R, Kurvers RA, van Wijk JA, Schreuder MF. Risk factors for renal injury in children with a solitary functioning kidney. *Pediatrics.* 2013;131(2):e478-485.

70. Whittam BM, Calaway A, Szymanski KM, et al. Ultrasound diagnosis of multicystic dysplastic kidney: is a confirmatory nuclear medicine scan necessary? *J Pediatr Urol.* 2014;10(6):1059-1062.

71. Yamamoto K, Kamei K, Sato M, et al. Necessity of performing voiding cystourethrography for children with unilateral multicystic dysplastic kidney. *Pediatric Nephrology.* 2019;34(2):295-299.

72. Zambaiti E, Sergio M, Baldanza F, Corrado C, Di Pace MR, Cimador M. Correlation between hypertrophy and risk of hypertension in congenital solitary functioning kidney. *Pediatr Surg Int.* 2019;35(1):167-174.

73. Calaway AC, Whittam B, Szymanski KM, et al. Multicystic dysplastic kidney: is an initial voiding cystourethrogram necessary? *Can J Urol.* 2014;21(5):7510-7514.

74. Calisti A, Perrotta ML, Oriolo L, Ingianna D, Miele V. The risk of associated urological abnormalities in children with pre and postnatal occasional diagnosis of solitary, small or ectopic kidney: Is a complete urological screening always necessary? *World J Urol.* 2008;26(3):281-284.

75. Shankar S, Hay E, Ray S. Routine micturating cystourethrogram for multicystic dysplastic kidneys: have we moved on? *Arch Dis Child.* 2018;103(11):1094-1095.
